# Extensive Cerebral Venous Sinus Thrombosis Post COVID-19 Vaccination

**DOI:** 10.7759/cureus.33637

**Published:** 2023-01-11

**Authors:** Lee Min Foo, Wan-Hazabbah Wan Hitam, Mohtar Ibrahim, Khairy Shamel Sonny Teo

**Affiliations:** 1 Department of Ophthalmology and Visual Science, School of Medical Sciences, Health Campus, Universiti Sains Malaysia, Kota Bharu, MYS; 2 Ophthalmology, Eye Clinic, Hospital Universiti Sains Malaysia, Kota Bharu, MYS

**Keywords:** cerebral venous sinus thrombosis (cvst), cerebral venous thrombosis (cvt), sars-cov-2, vaccination, covid-19

## Abstract

Extensive cerebral sinus thrombosis following severe acute respiratory syndrome coronavirus 2 (SARS-CoV-2) vaccination is rare. We report the case of a 42-year-old man who presented with a severe generalized headache that was not relieved by analgesics for nine days. It started four days after he received the third dose of BNT162b2 (BioNTech/Pfizer). He also complained of numbness at the back of the neck, vomiting, mild blurring of vision, and diplopia. The visual acuity (VA) in the right eye was 6/9 (improved to 6/7.5 with a pinhole) and 6/6 in the left eye. He was not able to abduct both eyes and noticed a double image at lateral gaze. Fundoscopy showed swollen optic discs with the presence of disc hemorrhages. A computed tomography venogram (CTV) of the brain showed loss of normal signal void with filling defects in the superior sagittal sinus, straight sinus, bilateral transverse sinuses, bilateral sigmoid sinuses, and bilateral internal jugular veins. The nasopharyngeal swab sample was negative for SARS-CoV-2. His platelet was normal (271x10^9^/L) and his coagulation profile was normal. Workup for connective tissue disease was negative. He was diagnosed with extensive cerebral vascular thrombosis post-vaccination. He received a one-week course of subcutaneous clexane, followed by oral anticoagulant treatment. After treatment, his headache was relieved, and the diplopia subsided. The venous thrombosis was partially resolved. Both the swollen optic discs improved, and his VA improved to 6/6 in both eyes.

## Introduction

As of December 2022, more than five million people in Malaysia have been diagnosed with coronavirus disease 2019 (COVID-19), and more than 36,000 people have succumbed to COVID-19 [1]. The development and use of the severe acute respiratory syndrome coronavirus 2 (SARS-CoV-2) vaccines represented a significant advance in the management of the COVID-19 pandemic. SARS-CoV-2 vaccines available in Malaysia are BNT162b2 (BioNTech/Pfizer), ChadOx1 (AstraZeneca), CoronaVac (Sinovac), and Ad5-nCoV (CanSino) [1]. 

To date, more than 28 million people in Malaysia have received at least one dose of the SARS-CoV-2 vaccine [1]. However, people are still hesitant to receive the SARS-CoV-2 vaccine as they are unsure about its safety and potential side effects. In addition to the usual side effects of fever, exhaustion, headache, and discomfort at the injection site, other more concerning side effects such as Bell's palsy, myocarditis and pericarditis, and thrombosis associated with thrombocytopenia have been reported [2]. Cerebral venous sinus thrombosis (CVST) is rare, but it is the most serious side effect reported after the SARS-CoV-2 vaccination. We highlight a rare case of extensive CVST with bilateral abducens nerve palsy following BNT162b2 (BioNTech/Pfizer) vaccination.

## Case presentation

A 42-year-old man with underlying liver hemangioma, presented with a severe generalized headache for nine days. The headache was not relieved by analgesics. It was associated with numbness at the back of the neck, vomiting, mild blurring of vision, and diplopia. He received his booster dose (third dose) of BNT162b2 (BioNTech/Pfizer), four days before the onset of symptoms. He had previously received two doses of the messenger RNA-based SARS-CoV-2 vaccine (BioNTech/Pfizer) without any complication. He had no history of recurrent headaches or trauma. He had no fever, seizure, slurred speech, or body weakness.

**Figure 1:**
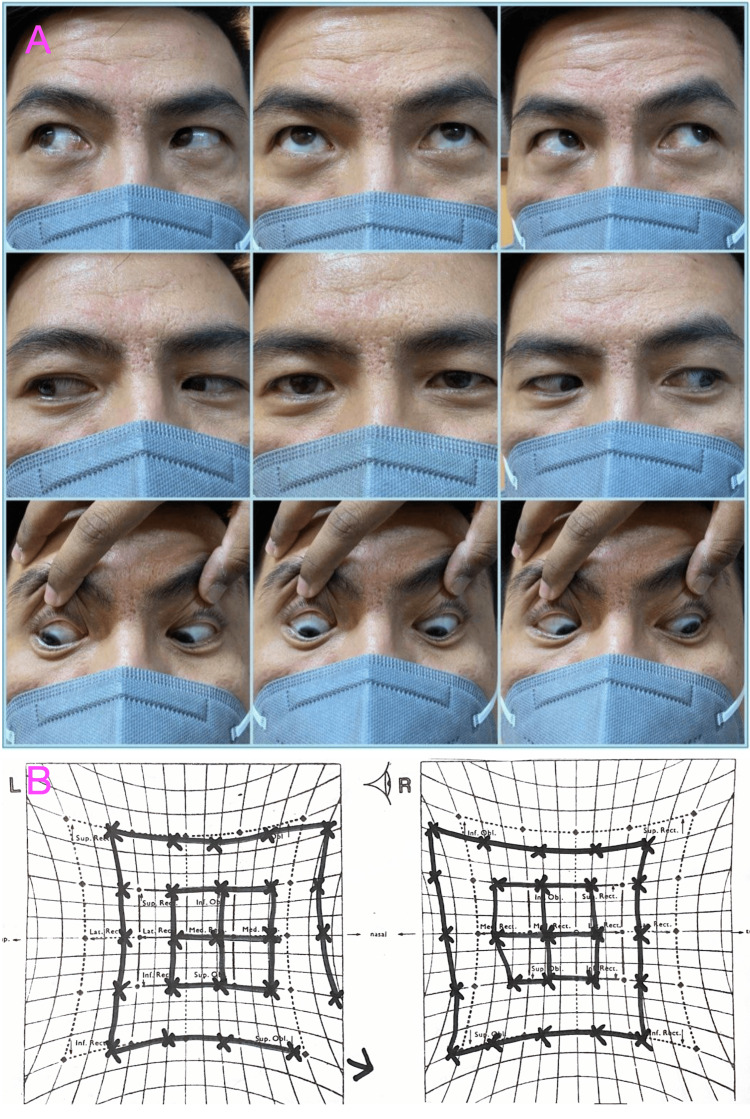
Nine cardinal gaze pictures (A) and Hess chart (B). A: Nine cardinal gaze pictures showing subtle limitations of abduction bilaterally; B: Hess chart showing limitation of abduction bilaterally.

**Figure 2:**
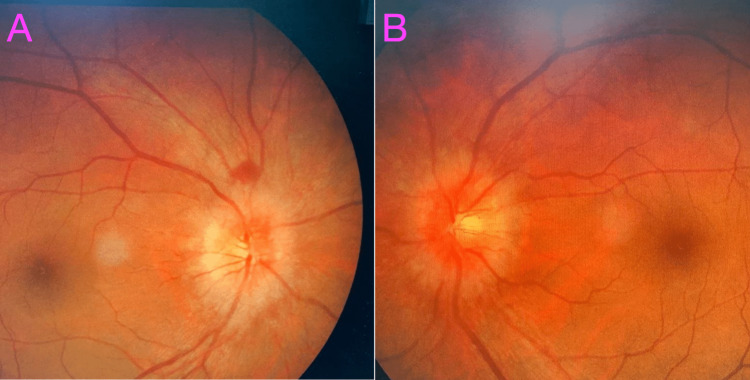
Fundus photos of both eyes. (A) Right eye showing swollen and hyperemic optic disc with disc hemorrhages. (B) Left eye showing swollen and hyperemic optic disc.

On ocular examination, visual acuity (VA) in the right eye was 6/9 which improved to 6/7.5 with a pinhole, and VA in the left eye was 6/6. The relative afferent pupillary defect was negative. He had subtle limitations of abduction bilaterally, as shown in Figures 1A-1B, and he had diplopia on the right and left gaze. Both anterior segments were unremarkable. Fundoscopy showed the right swollen and hyperemic optic disc with disc hemorrhages; the left eye optic disc was swollen and hyperemic (Figure 2). Both retinal vessels were normal with normal maculae. The neurological examinations were normal.

**Figure 3:**
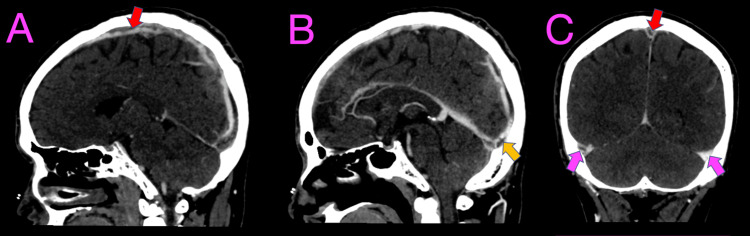
Computed tomography venogram of the brain. Computerized tomography venogram of the brain showing extensive thrombosis as filling defect in the superior sagittal sinus (A, C) (red arrows) with extension to straight sinus (B) (yellow arrow), and bilateral transverse sinuses (C) (pink arrows).

The nasopharyngeal swab sample was negative for SARS-CoV-2. His full blood count was normal, with a platelet count of 271 x 10^9^/L, and his coagulation profile was normal. Workup for connective tissue disease was negative. A computed tomography venogram (CTV) of the brain revealed a loss of normal signal void with filling defects in the superior sagittal sinus, straight sinus, bilateral transverse sinuses, bilateral sigmoid sinuses, and bilateral internal jugular veins, with no intracerebral hemorrhage (Figure 3). 30-2 Humphrey's visual field showed enlarged blind spots in both eyes.

He was diagnosed with extensive non-thrombosis-thrombocytopenia syndrome (TTS) CVST post-vaccination. He was treated with a one-week course of subcutaneous clexane, followed by oral apixaban 5 mg BD, oral Keppra 500 mg BD, oral piracetam 2.4 g BD, oral mecobalamin 500 mcg TDS, and analgesic. He was scheduled for a sequential follow-up. After treatment, his headache was relieved, his diplopia subsided, and his venous thrombosis partially resolved. His optic discs were no longer swollen, and his VA improved to 6/6 bilaterally.

## Discussion

In an analysis of 552 worldwide cases, De Gregorio et al. reported that most cases of CVST were associated with AZD1222/Vaxzevria vaccine (89.1%), followed by BNT162b2/CX-024414 Spikevax vaccine (8.2%), JNJ-78436735 vaccine (2.7%), and Covishield vaccine (0.3%) [3]. The cases were more common in women, with a women-to-men ratio of 2.25 to 1 [3]. In a systemic review and meta-analysis, Palaiodimou et al. reported that more than 70% of the CVST cases post-SARS-CoV-2 vaccination was linked to TTS, especially following vector-based vaccination [4]. On the other hand, non-vector-based vaccination was mostly related to non-TTS-CVST [4]. Similarly, in our case, our patient received a non-vector-based vaccination and subsequently developed non-TTS-CVST with a normal platelet count.

The pathogenesis of TTS following vector-based vaccination is known to be mediated by platelet factor 4 (PF4) reactive antibodies, which are induced by antigenic complexes of vaccine components [3-5], resembling spontaneous autoimmune heparin-induced thrombocytopenia (HIT) [6]. On the other hand, it is yet unknown what causes CVST after non-vector-based immunization. The hyper-inflammatory response brought on by mRNA binding to pattern recognition receptors may cause thrombus development [5]. Along with activating the alternative route, the mRNA also translates a spike protein that may promote platelet aggregation and dense granule production, enhancing IL-6 trans-signalling by activating the angiotensin II type 1 receptor [5].

In terms of clinical features, our patient resembles reported cases of non-TTS-CVST after BNT162b2 (BioNTech/Pfizer) vaccination, in that he is in his middle age (42 years old), with symptoms onset four days (reported cases with an interquartile range of 3-10 days) after vaccination [5]. Contrary to the reported cases, which developed CVST after the first (three cases) and second (four cases) vaccinations [5], our case developed CVST after his third vaccination. Thus, the absence of adverse effects after the first and second vaccinations does not exempt the patient from the danger of CVST for subsequent vaccinations.

For TTS-CVST, therapeutic anticoagulation with non-heparin products and intravenous immunoglobulin G (IVIG) is recommended as the first-line treatment [6,7]. In light of the resemblance between TTS-CVST and HIT and autoimmune HIT, heparin-containing anticoagulants should be avoided to prevent the progression of thrombosis after heparin use [6,7]. On the contrary, our case was treated with low-molecular-weight heparin (LMWH) followed by long-term direct oral anticoagulant (DOAC), as per practical guidelines for non-vaccine related CVST [8].

The outcome of CVST is generally favourable [8]. Compared to TTS-CVST, non-TTS-CVST was linked to a lower risk of mortality or dependency [3,4]. In similar reported cases of non-TTS-CVST after BNT162b2 (BioNTech/Pfizer) vaccination, three out of seven cases had residual neurological deficits [5]. On the contrary, our patient, despite having extensive CVST, had no neurological sequelae.

## Conclusions

The COVID-19 pandemic can still be fought most successfully using vaccines. A fourth dose of the SARS-CoV-2 vaccine is currently recommended for people who are more susceptible to COVID-19. Clinicians should be aware of the infrequent manifestation of CVST in patients who experience headaches or other neurological symptoms after non-vector-based vaccination, even though they had no adverse reactions to previous doses of vaccination. A better neurological prognosis for the patient may be possible with prompt diagnosis and early therapy commencement.
